# Tumor Detection at 3 Tesla with an Activatable Cell Penetrating Peptide Dendrimer (ACPPD-Gd), a T1 Magnetic Resonance (MR) Molecular Imaging Agent

**DOI:** 10.1371/journal.pone.0137104

**Published:** 2015-09-03

**Authors:** Christopher D. Malone, Emilia S. Olson, Robert F. Mattrey, Tao Jiang, Roger Y. Tsien, Quyen T. Nguyen

**Affiliations:** 1 Department of Radiology, University of California, San Diego, San Diego, CA, United States of America; 2 Howard Hughes Medical Institute, University of California, San Diego, San Diego, CA, United States of America; 3 Department of Pharmacology. University of California, San Diego, San Diego, CA, United States of America; 4 Department of Chemistry and Biochemistry, University of California, San Diego, San Diego, CA, United States of America; 5 Department of Otolaryngology-Head and Neck Surgery University of California, San Diego, San Diego, CA, United States of America; Universidade de São Paulo, BRAZIL

## Abstract

**Purpose:**

The ability to detect small malignant lesions with magnetic resonance imaging (MRI) is limited by inadequate accumulations of Gd with standard chelate agents. To date, no T1-targeted agents have proven superiority to Gd chelates in their ability to detect small tumors at clinically relevant field strengths. Activatable cell-penetrating peptides and their Gd-loaded dendrimeric form (ACPPD-Gd) have been shown to selectively accumulate in tumors. In this study we compared the performance of ACPPD-Gd vs. untargeted Gd chelates to detect small tumors in rodent models using a clinical 3T-MR system.

**Materials and Methods:**

This study was approved by the Institutional-Animal Care-and-Use Committee. 2 of 4 inguinal breast fat pads of 16 albino-C57BL/6 mice were inoculated with tumor Py8119 cells and the other 2 with saline at random. MRI at 3T was performed at 4, 9, and 14 days after inoculation on 8 mice 24-hours after injection of 0.036mmol Gd/kg (ACPPD-Gd), and before and 2–3 minutes after 0.1 mmol/kg gadobutrol on the other 8 mice. T1-weighted (T1w) tumor signal normalized to muscle, was compared among the non-contrast, gadobutrol, and ACPPD-Gd groups using ANOVA. Experienced and trainee readers blinded to experimental conditions assessed for the presence of tumor in each of the 4 breast regions. Receiver operator characteristic (ROC) curves and area-under-curve (AUC) values were constructed and analyzed.

**Results:**

Tumors ≥1mm^3^ were iso-intense to muscle without contrast on T1w sequences. They enhanced diffusely and homogeneously by 57±20% (p<0.001) 24 hours after ACPPD-Gd and by 25±13% (p<0.001) immediately after gadobutrol. The nearly 2-fold difference was similar for small tumors (1-5mm^3^) (45±19% vs. 19±18%, p = 0.03). ACPPD-Gd tended to improve tumor detection by an experienced reader (AUC 0.98 vs 0.91) and significantly more for a trainee (0.93 vs. 0.82, p = 0.02) compared to gadobutrol. This improvement was more pronounced when obvious tumors (>5mm^3^) were removed from the ROC analysis for both the experienced observer (0.96 vs. 0.86) and more so for the trainee (0.86 vs. 0.69, p = 0.04).

**Conclusion:**

ACPPD-Gd enhances MMP-expressing tumors of any size at 3T 24 hours after administration, improving their detection by blinded observers when compared to non-contrast and contrast groups given commercial Gd-chelates and imaged during the equilibrium phase.

## Introduction

Early and accurate detection of malignant lesions is essential for complete eradication [1]. Contrast-enhanced magnetic resonance (MR) imaging with T1-shortening gadolinium (Gd)-chelates plays an important role in detecting tumors relying on their pattern, rate, and degree of enhancement. Although these patterns improve characterization, MRI remains limited with high false positive rates [2, 3].

Numerous T1 or T2 MR molecular imaging agents have been proposed to improve detection and characterization of malignant lesions. Many of these exhibit a tropism for an up-regulated molecule or extracellular milieu unique to malignancies, such as estrogen receptor (ER) [4], epidermal growth factor receptor (EGFR) [5], tyrosine phosphatase (PTPμ) [6], and matrix metalloproteinases (MMPs) [7,8], among others. Their major challenge remains the poor sensitivity of MR to the amount of reporter that accumulates within tumors to achieve sufficient contrast-to-noise-ratio relative to background [9, 10].

MMPs are a family of proteases predominantly involved in extracellular matrix breakdown. They promote tumor spread and are associated with more aggressive tumors, including the breast triple negative subtype [11–16]. Therefore, tumor-MMPs are valuable markers for not only detection, but also for recognizing aggressive tumors.

**Fig 1:**
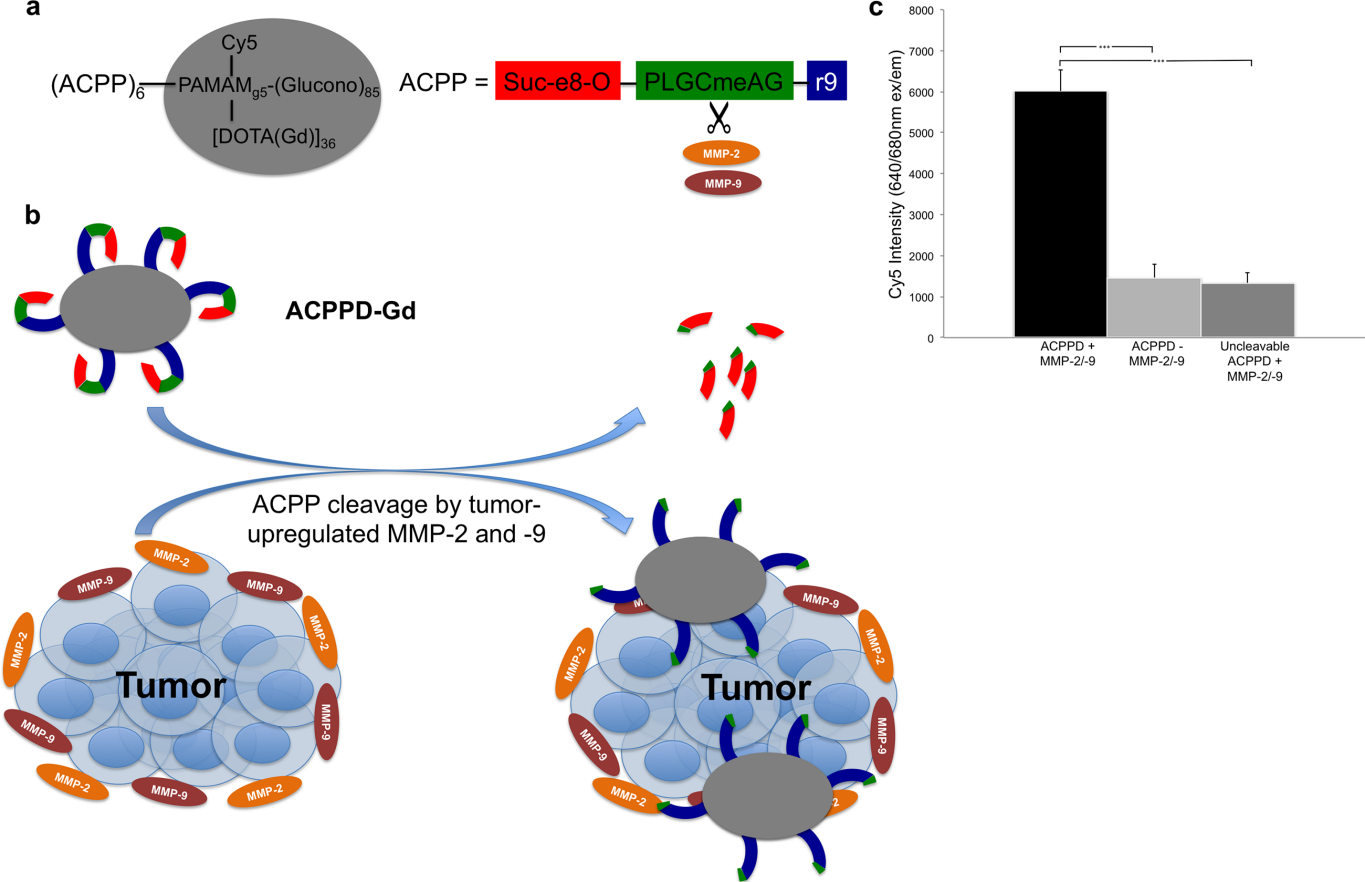
Overview of ACPPD-Gd structure and function. **a**, The overall structure of the ACPPD-Gd dendrimer as previously described (20). Here, the payload consists of 36 Gd-DOTA and 1 Cy5 molecules for dual MR and fluorescence imaging. The PLGCmeAG peptide linker is cleaved in the presence of the proteases MMP-2 and -9, which are upregulated in tumors. **b**, After tumor protease-dependent cleavage of the ACPP linker, the polycation and polyanion peptides separate allowing the highly cationic polyarginine peptide, linked to the dendrimer with payload (Gd-DOTA and Cy5), to bind to tumor cells and stroma. **c**, ACPPD-Gd is preferentially cleaved and binds to Jurkat cells in the presence of recombinant MMP-2 and MMP-9 *in vitro*. The dendrimer used in our study demonstrated significantly higher binding to Jurkat cells *in vitro* after overnight incubation with MMP-2 and -9 as measured by Cy5 fluorescence, compared to dendrimer incubated without MMPs and an uncleavable dendrimer (PLGCmeAG replaced by hexa(ethyleneoxy)) incubated in the presence of MMPs under the same conditions. *** indicates p<0.001.

Activatable cell-penetrating peptides (ACPPs) consist of a polycationic peptide attached to a charge neutralizing polyanion via a protease-cleavable linker, in this case an MMP cleavable linker. While this linker is cleaved to some extent by a variety of MMPs, it is most specific for MMP-2 and MMP-9 (MMP-2/-9) [17]. When the linker is cut, the polycation that carries the Gd binds to and/or becomes internalized by surrounding cells, thereby increasing local Gd concentration (Fig 1a and 1b) [17–20]. The agent used in this study is a 5th-generation PAMAM dendrimer decorated with multiple ACPP and Gd-DOTA molecules (ACPPD-Gd), which specifically enhanced tumors at 7T [20]. The dendrimer carrier both increases the in vivo half-life of the molecule as well as increases the tumor uptake of gadolinium by 4 to 15 fold as compared to the free peptides, though at the expense of selectivity (four-fold for the free peptide vs. two-fold for the conjugated ACPP’s) [20]. The preferential uptake of cleavable ACPPD-Gd versus D-amino acid controls has been validated not only in other MMP-expressing tumor models [20], but also in acute stroke, where MMP-2/9 have also been shown to be overexpressed [21]. In addition, we have validated this *in vitro*, as the ACPP attached to dendrimers used in our study are preferentially cleaved by recombinant MMP-2 and -9 resulting in several fold increased binding to Jurkat cells compared to when MMP-2/-9 are absent or an uncleavable ACPP is exposed to MMP-2/-9 (Fig 1c).

Several MMP-2/9 sensitive molecular imaging agents have been made with similar selectivity [7,8,20,22]. However despite the multiple attempts at imaging MMP-2/9, it is unclear whether imaging these molecules offers any potential clinical advantage in detecting smaller tumor sizes than commercial Gd chelates. ACPPD labeled with Cy5 have already demonstrated a substantial benefit in tumor resection performed with fluorescence guidance with added survival times and increased negative margin resections in tumor-bearing mice [23]. We hypothesized that these agents linked to Gd would add a similar “clinical” benefit in animals by increasing tumor detection capabilities versus the current standard clinical Gd-chelate agents. Here we have taken the first step toward answering that question by injecting MMP-2/-9 producing tumor cells or control saline into the mammary fatpads of mice and determining whether the ACPPD-Gd agent offers an advantage over commercial Gd chelates for detecting tumors of varying sizes. Tumors were injected into the fatpad in a blinded fashion and examined longitudinally. This is analogous to clinical radiologic imaging, in which tumors “detected” early are often followed by imaging. Animals were imaged at clinical field strength (3T), where inherent signal-to-noise-ratio (SNR) and spatial resolution are lower than at 7T. As a control, we have chosen the small molecule Gd-DO3A-butrol (gadobutrol), a commercial Gd chelate frequently used for clinical oncologic applications. We found that the MMP-2/9 sensitive agent improved radiologist performance relative to the small molecule gadolinium agent and no injection.

Although MRI is currently not a screening test for tumor detection, this is partly due to low sensitivity/specificity of the currently available non-targeted contrast agents. In this study, we show that ACPPD-Gd has higher sensitivity/specificity compared to untargeted Gd chelates in a clinical 3T-MRI, suggesting that it may be useful as a screening tool in selected patient population.

## Materials and Methods

### Contrast Agent

ACPPD was synthesized according to previously reported methods [20] with minor modifications. Briefly, peptide Suc-e_8_-(Aop)-PLG-C(Me)-AG-r_9_-c-NH_2_ (Suc = succinyl, e = d-glutamate, Aop = 5-amino-3-oxapentanoyl, C(Me) = S-methylcysteine, P = proline, L = leucine, G = glycine, A = alanine, r = d-arginine, c = d-cysteine) was synthesized by standard Fmoc chemistry and purified on HPLC. Six copies of peptides were then conjugated via their C-terminal cysteines to a generation 5 poly(amidoamine) dendrimer via maleimide linkers. 36 DOTA and 1 Cy5 were then attached to the dendrimer through amide linkages. The remaining free amine groups on the dendrimer were capped by reaction with a large excess of δ-gluconolactone in the presence of N-methylmorpholine for 3 days at room temperature. MethoxyPEG_4_-N-hydroxysuccinimide was added at the last step to ensure capping of any leftover free amine groups on the dendrimer. The product was purified by membrane filtration with an Amicon centrifugal filter (30KDa cutoff). Gd was chelated onto DOTA on the ACPPD by heating it with glycine buffer (pH 6) and GdCl_3_ at 40°C overnight. The number of Gd on ACPPD was measured as described under **Post mortem analysis**. Molecular weight of the final dendrimer was approximately 29kDa, with a R1 relaxivity of 3.7(mM Gd)^-1^s^-1^ at 7 T [20].

### *In vitro* Jurkat assay

The cleavable ACPPD used in this study and an uncleavable version (linker peptide replaced by multiple PEG6 groups) were incubated overnight with and without 3.5μL MMP-2 and -9 cocktail at 37°C in Tris (20 mM) normal saline, 2 mM CaCl_2_, pH 7.5 buffer. Final dendrimer concentration was 5 μM. The dendrimer/MMP mix was divided into triplicate samples and incubated with Jurkat cells (4x10^6^cells/ml) at 37°C for 15–20 minutes. Samples were spun down and washed with Hank’s balanced salt solution (HBSS) 3 times, and the final pellet was resuspended in 100μL HBSS. Final resuspensions were assayed for Cy5 intensity on a 96-well plate reader at an excitation/emission (ex/em) of 640/680nm.

### Animal Model

The University of California San Diego Institutional Animal Care and Use Committee (IACUC) approved this research under protocol S04011. All steps were ensured to minimize pain and suffering of all animals during the course of this experiment. Anesthesia during tumor inoculation and MR imaging was isoflurane/O_2_. All animals were given free access to food and water *ad libitum* during the entire study. Euthanasia involved CO_2_ asphyxiation followed by cervical dislocation. Each of the 4 inguinal nipples of 16 albino C57BL/6 (Harlan Labs) mice were randomized such that each mouse had 2 mammary fat pads inoculated with 10^4^ Py8119 mammary tumor cells [24] and the other 2 with an equal volume of saline. Py8119 cells were derived from the transgenic polyoma middle-T spontaneous tumor model that can be transplanted to reliably form tumors, and demonstrate significantly increased MMP-9 expression compared to the more indolent luminal cell lines. This cell line is also considered triple-negative. They are syngeneic in the C57Bl/6 strain, enabling a robust response from normal immune cells.

### Imaging

All mice were imaged on days 4, 9, and 14 after inoculation under isoflurane/O_2_ anesthesia. Imaging the same mouse 3 times provided a wide range of tumor sizes while minimizing animal utilization. Each mouse was randomized to receive IV either ACPPD-Gd (20 nmol, 0.036mmol Gd/kg) (8 mice) or gadobutrol (0.1mmol/kg, Gadavist, Bayer HealthCare Pharmaceuticals Inc.) (8 mice) for all three imaging sessions. Imaging was performed 24 hours after ACPPD-Gd, and before and 2–3 minutes after gadobutrol. The pre-contrast study served as a non-contrast control group, resulting in 8 mice imaged without contrast, 8 at equilibrium phase following gadobutrol, and 8 24 hours following ACPPD-Gd. Optimal ACPPD-Gd dose and imaging time were previously determined [20].

MRI was performed on a clinical 3T Signa HDx TwinSpeed scanner (GE Healthcare Technologies, Milwaukee, WI) using a transmit/receive 2.5cm finger coil. Four Fast Spin-echo pulsing sequences with 6 echo-train-length were all acquired with 256x256 matrix, FOV 6.0x4.2cm, 0.9mm slice thickness (Voxel size 0.23x0.16x0.9mm), and NEX = 1. Two T1-weighted axial and coronal (TR/TE = 700/8.3ms, BW = 50KHz, Acq. time = 8min, 47s total), and fat saturated axial T1 (TR/TE = 500/8.5, BW = 42KHz, Acq. time = 5min, 33s) and T2 (TR/TE = 6800/81.8,BW = 15.63KHz, Acquisition time = 3min, 31s) were acquired. All 4 sequences were acquired pre as well as post gadobutrol injection for all 3 time points.

### Image Analysis

Tumor signal and size were quantified at each time point using region-of-interest (ROI) analysis with OsiriX software (Pixmeo, Geneva). If a mouse had two tumors, each tumor was treated independently. Tumors <1mm^3^ were not analyzed because of unreliable ROI placement and partial volume effects. A polygonal ROI was traced around the tumor on the fat-saturated T1-weighted axial sequence at its maximal dimension and the average signal and cross-sectional area recorded. The signal of an adjacent muscle on the same slice was measured with a comparable ROI and tumor signal normalized as [(tumor signal – muscle signal)/muscle signal]. Tumor volume was calculated using an ellipsoid function as 4/3*(area of tumor ROI)*(1/2)*(maximal orthogonal coronal diameter). Normalized liver signal – average of ≥5 ROI’s on the coronal slice, was similarly calculated relative to adjacent muscle.

The acquired 72 studies (3 groups x 8 mice x 3 time points) with 4 pulsing sequences each were anonymized and presented at random to 2 observers blinded to contrast grouping, time since inoculation, and which 2 nipples were inoculated with tumor. Reader 1 is an MR expert with extensive experience in clinical and animal MR image interpretation (RFM) and Reader 2 is a 2^nd^-year radiology resident in training (ESO). Studies were reviewed independently and readers reported whether the breast tissue under each of the 4 nipples in each study was normal or contained tumor along with their confidence level as low, medium, or high. If a tumor was present they described the degree and pattern of tumor enhancement.

### Post mortem analysis

Mice were sacrificed immediately after the 14-day MRI study and tumors, liver, kidneys, and muscle were harvested and immediately frozen at -20°C for later analysis. Tissue Gd content was measured using inductively coupled plasma-mass spectrometry (ICP-MS) on defrosted and weighted tissues. Less than 100mg samples of each tissue were placed in 900uL of nitric acid on a Gyromixer overnight at room temperature. The next day, 200μL of 30% hydrogen peroxide were added, and after 2 hours the samples were heated to 90–100°C for 2 hours. Tuning solution was then added to a 5mL total volume. Samples were then run in an Agilent Technologies 7700 Series ICP-MS to measure gadolinium content in parts-per-billion. Tissue Gd concentration in micromolar was calculated as (ppb of Gd/Gd molecular weight)*(total weight of solution measured/tissue wet weight).

### Data Analysis

Each contrast group was subdivided into 3 tumor size groups (1–4.9mm^3^, 5–14.9mm^3^, and ≥15mm^3^) and the difference in normalized tumor intensities was analyzed for statistical significance using a 3-way analysis of variance (ANOVA) without interactions (MATLAB (MathWorks, Natick, MA), where contrast group, tumor size, and time after tumor inoculation served as the independent variables. Statistical significance was assumed when the 2-tailed unpaired Student’s t-test comparing the ACPPD-Gd and the other 2 groups, and the paired t-test comparing the non-contrast to the gadobutrol group was <0.05. Data are presented as mean±standard deviation.

Receiver operating characteristic (ROC) curves were constructed using 4 levels: normal, low, medium and high probability for tumor presence. There were 288 breasts evaluated (3 groups x 8 animals x 3 time-points x 4 breasts). Breasts that had been inoculated with Py8119 cells, but did not have a tumor on postmortem, were counted as normal. Additional ROC curves were constructed for tumors <5mm^3^ to assess the potential benefit of ACPPD-Gd in detecting small tumors. Interobserver agreement was assessed for all 288 breasts and within each of the 3 contrast groups using the kappa statistic [25]. Area under the ROC curve (AUC) was calculated for the 3 contrast groups using the JROC web-based program [26, 27]. The bootstrap method was used to compute 95% confidence intervals for the AUCs and to compare AUC of two-paired or unpaired ROC curves [28]; 2000 bootstrapped replicates were used for each comparison and the analysis was performed using the pROC package in R software (version 3.0.2, www.r-project.org).

## Results

### Tumor Size

Imaging mice 3 times after inoculation provided a range of tumor sizes from 0.03 to 107.4 mm^3^ (~0.4 to 5.9mm in diameter). Of the 144 potential tumor-containing mammary fad pads imaged (3 groups x 8 mice x 2 tumors x 3 time points), a few tumors either did not grow or were only seen at later time points resulting in 107 tumors, 39 in the ACPPD-Gd group, and 34 in each of the non-contrast and gadobutrol groups. Number of tumors distributed by size and contrast group is shown in Table 1.

**Table 1:** Distribution of Tumor Size for each Contrast Group.

	<1mm^3^	1-5mm^3^	5-15mm^3^	>15mm^3^	Total
ACPPD-Gd	10	10	7	12	39
Non-Contrast	15	4	6	9	34
Gadobutrol	15	4	6	9	34

### Tumor enhancement

**Fig 2:**
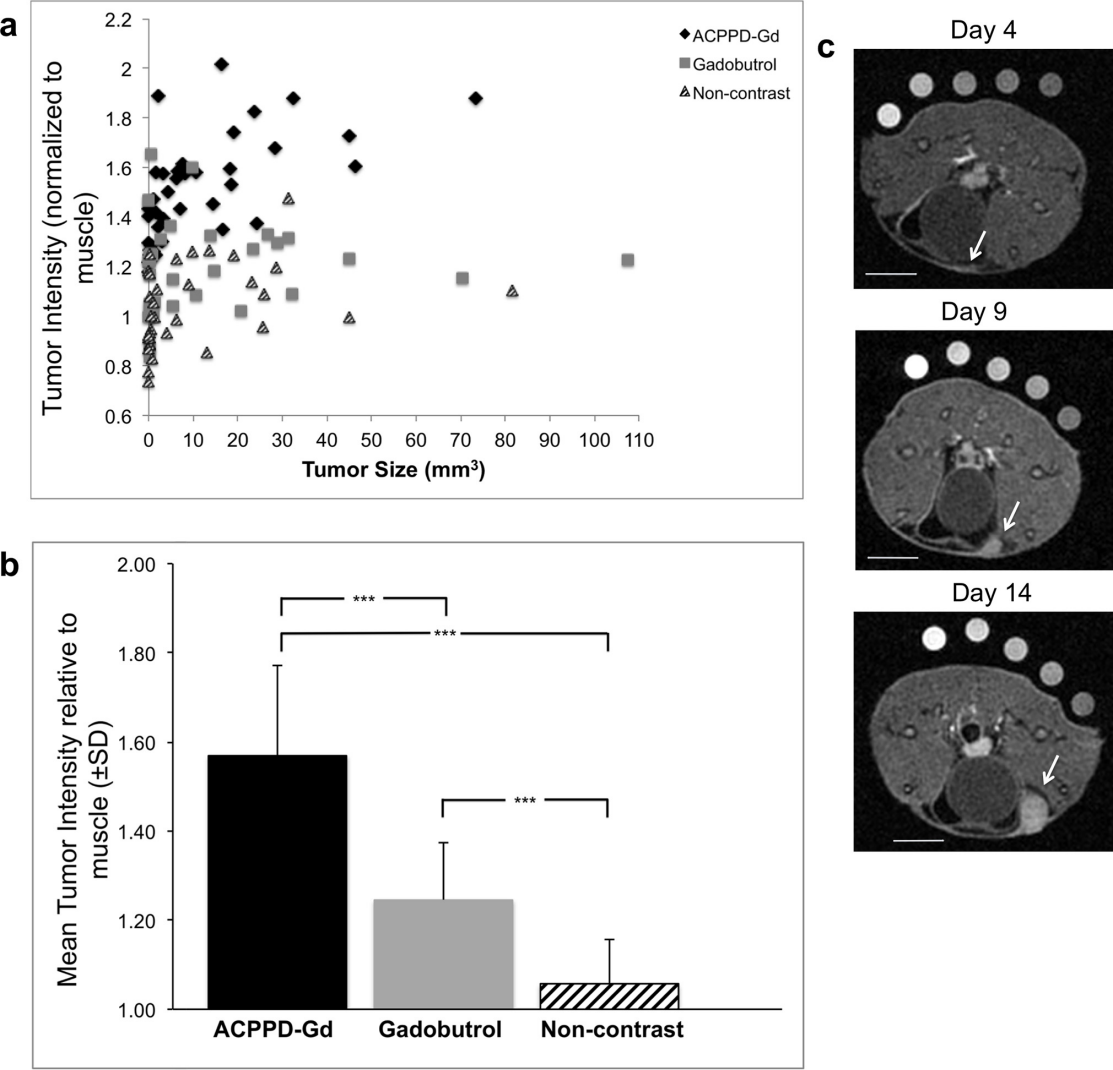
Tumor size and enhancement of all tumors. **a**, The normalized signal of all 107 tumors is shown as a function of tumor size that ranged from 0.03 to 107.4 mm^3^. Note that tumor enhancement was consistently greater for ACPPD-Gd animals at all tumor sizes. **b**, Bar graph of normalized mean enhancement ± SD of tumors >1mm^3^ shows that ACPPD-Gd caused the greatest enhancement. *** indicates p<0.001. **c**, Representative axial fat-saturated T1w MR images of a tumor bearing mouse given ACPPD-Gd and imaged on days 4, 9, and 14 after inoculation showing a thin strip of enhancement on day 4 (arrow) at the site where the tumor became apparent on days 9 and 14 (arrow). This was not observed in the gadobutrol group (not shown). Scale bars = 5mm for each image.

**Fig 3:**
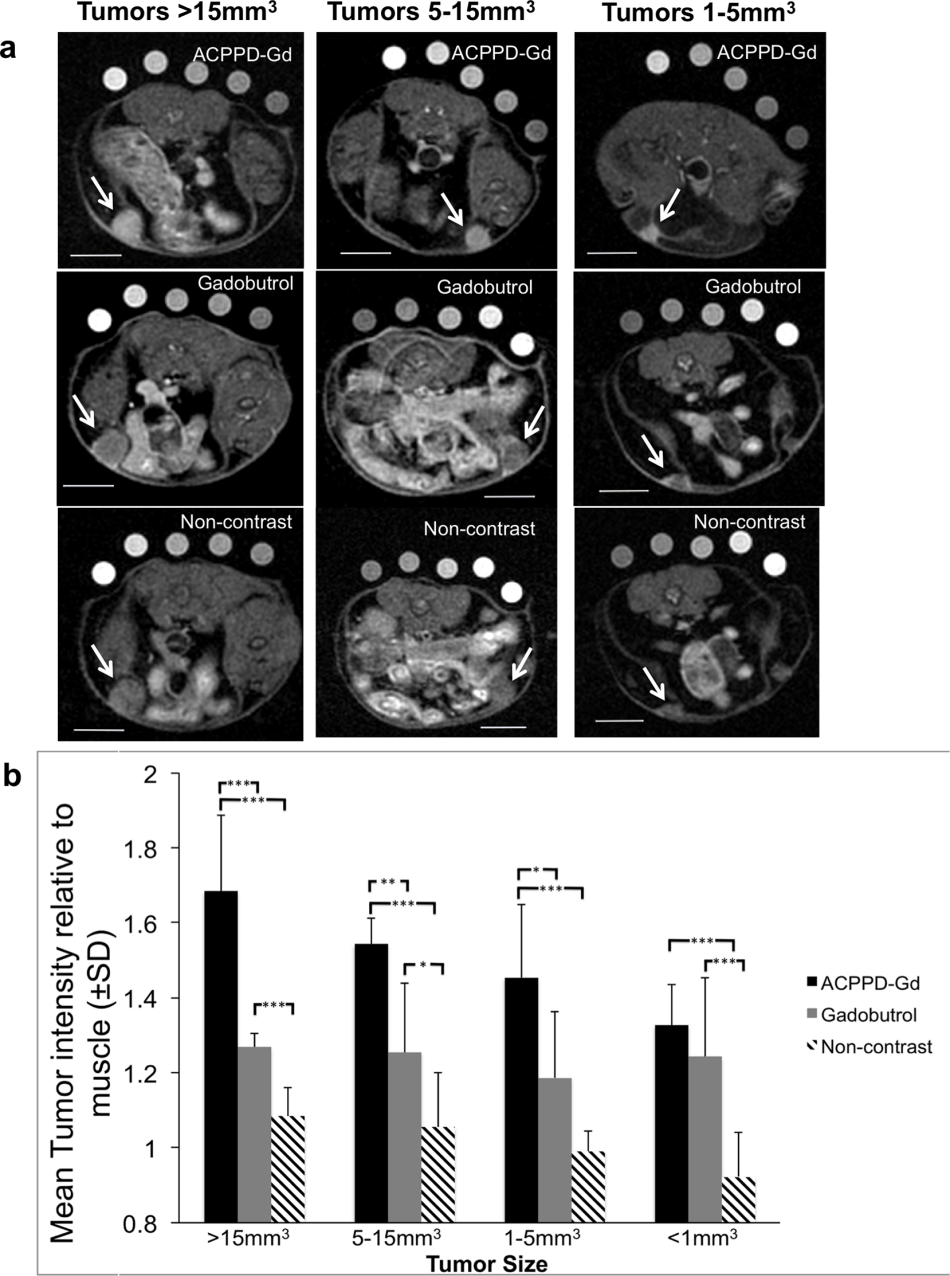
Degree and pattern of enhancement as a function of tumor size. **a**, Representative axial fat-saturated T1w MR images of mice given ACPPD-Gd (top row) and others imaged before (bottom row) and after gadobutrol (middle row) with comparable size tumors shown with the same window and level. Note that while ACPPD-Gd produced homogenous diffuse tumor enhancement (arrows), gadobutrol enhanced predominantly the tumor rim. Scale bar in each image = 5mm. **b**, Bar graph shows mean tumor enhancement ± SD for each contrast group as a function of tumor size groups. * indicates p<0.05, **p<0.01, ***p<0.001.

Degree of tumor Intensity relative to muscle for all 107 tumors is shown in Fig 2a; mean relative intensity and standard deviation of those tumors ≥1mm^3^ are shown in Fig 2b. Note that while pre-contrast tumors (n = 19) were essentially iso-intense to muscle (1.06±0.10), they enhanced by 24.8%±12.8, p<0.001, 2–3 minutes after gadobutrol injection. Tumors ≥1mm^3^ (n = 29) imaged 1 day after ACPPD-Gd, enhanced by 49.0%±20.0 relative to the non-contrast group, p<0.001, and 26.0% greater than the gadobutrol group, p<0.001 (Fig 2b). ACPPD-Gd administration outlined a very small strip of enhancement at the earliest time point in breasts destined to develop tumors (Fig 2c). This strip of enhancement was not seen at control saline injection sites. Representative images of pre and post-gadobutrol and those imaged 24 hours after ACPPD-Gd are shown in Fig 3a. Note that ACPPD-Gd produced diffuse homogenous tumor enhancement, while gadobutrol enhancement was more apparent at the tumor periphery. Tumor enhancement with gadobutrol was independent of tumor size (Fig 3b). Tumors as small as 1-5mm^3^ showed significantly greater enhancement following ACPPD-Gd as compared to gadobutrol (45±19% vs. 19±18%, p = 0.03).

### Effect of ACPPD-Gd on Observer Performance Characteristics

**Fig 4:**
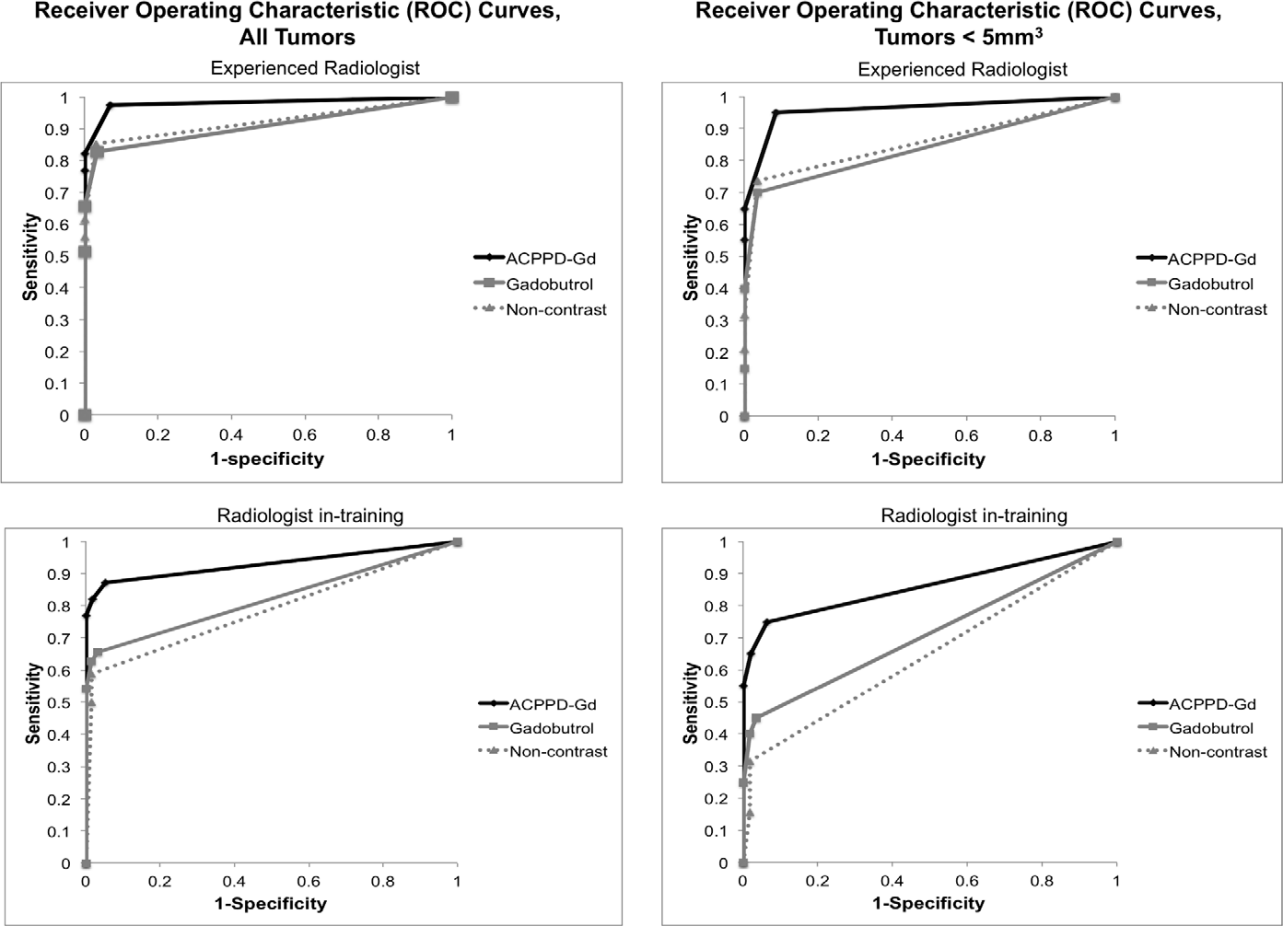
ROC curves. The ROC curves are plotted for the experienced (top row) and trainee (bottom row) for all 288 breasts (left column) that were normal (n = 181) or tumor bearing (107) and the breasts with small tumors (<5mm^3^) (n = 58) and the associated normal breasts within the same animals (right column). Note that the AUC improved for both observers after ACPPD-Gd but the improvement was more significant for the trainee.

The performance of the experienced and trainee readers reflected their MR experience. The AUC after gadobutrol administration relative to the non-contrast group was identical for the experienced observer at 0.92, and minimally improved but was not statistically significant for the trainee (0.81 vs. 0.79; p = 0.5) (Fig 4). Following ACPPD-Gd, the AUC increased from 0.92 to 0.98, (p = 0.08) for the experienced observer and from 0.81 to 0.93, (p = 0.02) for the trainee (Table 2). The improvement in the trainee’s performance after ACPPD-Gd was even more pronounced when obvious tumors (>5mm^3^) were removed from the ROC analysis with AUC values of 0.69 (gadobutrol) and 0.86 (ACPPD-Gd), p = 0.04 (Table 3) (Fig 4). Inter-observer agreement was very good (Cohen’s weighted kappa = 0.80) when comparing readings from all 3 contrast groups; however, inter-observer agreement was greater for the ACPPD-Gd (kappa = 0.83), than the gadobutrol groups (kappa = 0.74).

**Table 2:** AUCs (95%CIs) from ROC curves for classifying tumor presence status based on tumor presence confidence scores given by group and reader for all tumors (n = 107).

Group	Reader 1 *	Reader 2 *
ACPPD-Gd	0.98 (0.95, 0.99)	0.93 (0.87, 0.99)
Gadobutrol	0.92 (0.86, 0.98)	0.81 (0.73, 0.90)
Non-contrast	0.92 (0.85, 0.98)	0.785 (0.70, 0.87)
p-value, ACPPD-Gd vs. Gadobutrol	p = 0.08	p = 0.02

* Comparisons between interpreters: ACPPD-Gd, p = 0.04; gadobutrol, p = 0.003; non-contrast, p = 0.0003.

**Table 3:** AUCs (95%CIs) from ROC curves for classifying tumor presence status based on tumor presence confidence scores given by group and reader for all tumors <5mm^3^ (n = 58).

Group	Reader 1 *	Reader 2 *
ACPPD-Gd	0.96 (0.89, 0.99)	0.86 (0.75, 0.95)
Gadobutrol	0.86 (0.75, 0.96)	0.69 (0.57, 0.81)
Non-contrast	0.85 (0.75, 0.95)	0.66 (0.55, 0.76)
p-value, ACPPD-Gd vs. Gadobutrol	0.10	0.04

* Comparisons between interpreters: ACPPD-Gd, p = 0.047; gadobutrol, p = 0.003; non-contrast, p = 0.0007.

### Effect of repeat injections of ACPPD-Gd

**Fig 5:**
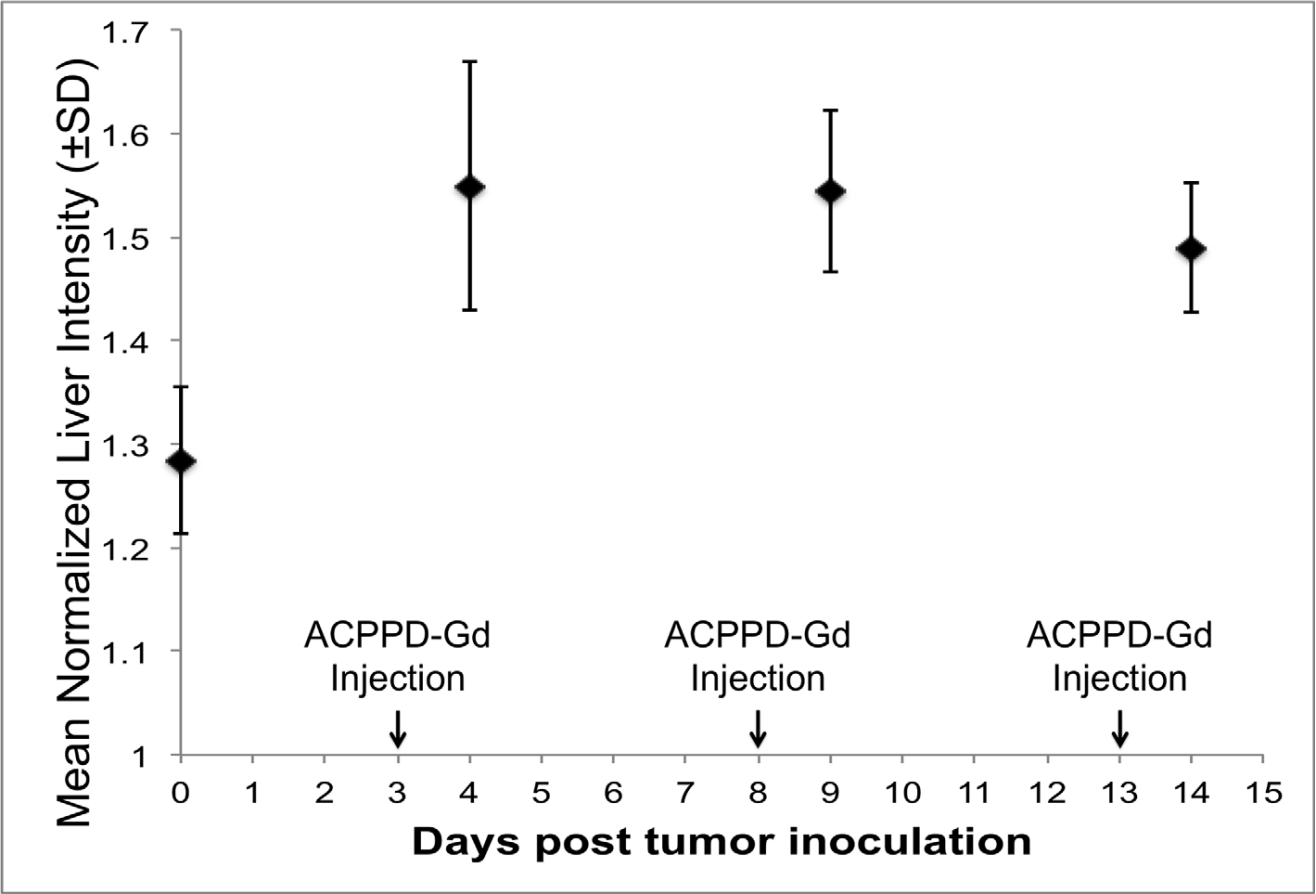
Effect of cumulative ACPPD-Gd injections on Liver Enhancement. Mean liver enhancement ± SD as measured on T1w coronal scans normalized to muscle just prior to tumor inoculation and before any ACPPD-Gd administration (day 0) and 24 hours following ACPPD-Gd on days 4, 9, and 14. Note that liver enhancement of approximately 25% greater than baseline liver signal remained similar after each injection.

To ensure that each imaging session can be treated independently with no residual ACPPD enhancement remaining 5 days after injection as reported, we evaluated liver signal where the greatest ACPPD-Gd accumulation occurs [17–20]. The 23% greater liver enhancement of the ACPPD group relative to the non-contrast group was similar on days 4, 9, and 14 indicating that there was no cumulative effect 5 days after ACPPD administration (Fig 5).

### Gd Tissue Concentration

**Fig 6:**
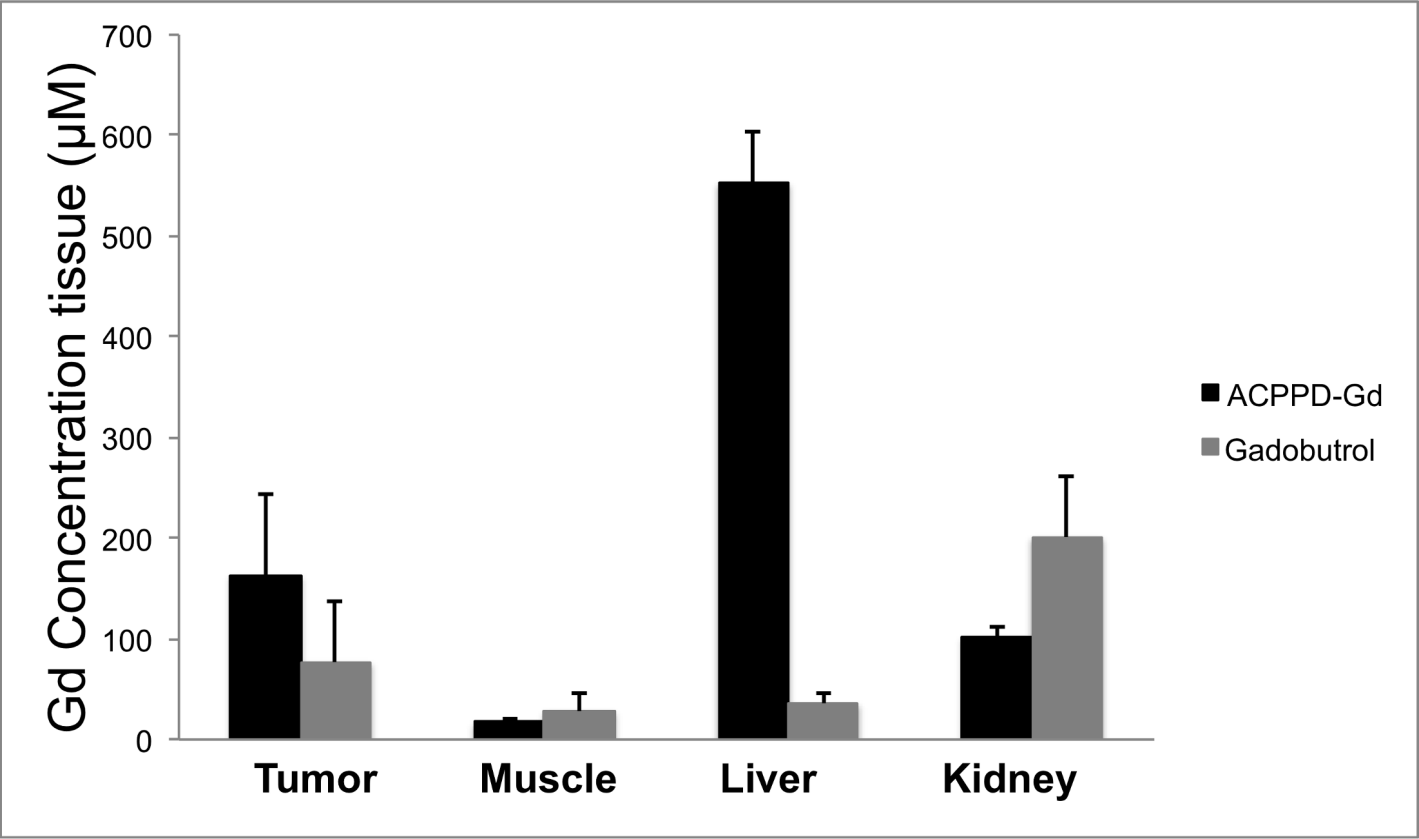
Tissue concentrations following ACPPD-Gd and gadobutrol. ACPPD-Gd resulted in significantly greater accumulation of Gd in tumors (163 ± 81 vs. 77±60 μmole Gd/mg tissue, p<0.01) than gadobutrol and resulted in approximately 3.5 times greater tumor to muscle concentration ratio (9.3 ± 0.5 vs. 2.7 ± 1.0).

As observed with imaging, tumor Gd concentration at 14 days after inoculation was significantly greater 24 hours after the administration of 0.036 mmol Gd/kg ACPPD-Gd than after 0.1mmol Gd/kg of gadobutrol at equilibrium phase (163 ± 81 vs. 77±60 μmole Gd/mg tissue, p<0.01) (Fig 6). More important for image contrast, ACPPD-Gd produced nearly 3.5 times greater tumor to muscle concentration ratio than gadobutrol (9.3 ± 0.5 vs. 2.7 ± 1.0) (Fig 6). The Gd biodistribution after administration of the gadobutrol at equilibrium phase was typical for a small molecular weight agent, where the majority of Gd was in the kidneys. In contrast, 24 hours after ACPPD-Gd administration the dominant accumulation was in the liver. The exact location of ACPPD-Gd accumulation within the liver and whether ACPPD-Gd was cleaved was not investigated in this study.

## Discussion

These results demonstrate the added benefit of a molecularly targeted T1 agent in the detection of MMP expressing tumors at 3 Tesla. Tumor enhancement at 24 hours was 3.5 times greater after the administration of 36% the Gd dose of a standard chelate imaged at equilibrium. ACPPD-Gd enhanced tumors homogenously as compared to the typical rim enhancement observed with Gd chelates. This in part impacted tumor enhancement values since the ROI was placed over the entire tumor, but also decreased tumor conspicuity particularly for the upper two nipples where tumors were adjacent to bowel that appeared similar to ring enhancing tumors in this animal model (Fig 3a).

Unlike the rapid leak of Gd-chelates across normal and particularly abnormal capillaries, ACPPD-Gd circulates in blood with a half-life of 9 hours [20], preferentially leaking across abnormal capillaries to interrogate the interstitial space for the presence of MMP-2/9. When cleaved, the ACPP promotes local trapping, increasing local Gd concentration and tissue enhancement. It is likely that the delivery of ACPPD nanoparticles shortly after intravenous administration is predominantly to the periphery of the tumor where blood flow is highest and where the leaky capillaries predominate [29]. Previous experiments suggest that approximately 50% of uptake is due to the MMP-2/9 based mechanism and 50% is due to EPR, and this is compatible with our current results as well. The measured ACPPD concentration of 163μM is much greater than the 50μM previously reported [20], which could be due to 1) the replacement of most methoxyPEG_4_ groups by the extremely hydrophilic gluconamides as capping groups, leading to increased solubility and decreased tendency for hepatobiliary excretion; 2) the larger Gd load per dendrimer; or 3) least likely, the cumulative accumulation of ACPPD. These changes are small and given similar uptake patterns in an ex vivo cell-based assay, we presume that the new molecule has similar MMP-2/9 sensitivity, with approximately 50% of overall uptake being caused by the MMP mechanism and the remaining 50% being due to enhanced permeability and retention as well as blood pool.

While ACPPD-Gd is relatively selective for MMP-2 and MMP-9, it is a generic contrast agent to any tissue that overexpresses those MMPs, particularly cancer, since MMPs are abundantly expressed in virtually all tumor lines during various stages of malignant progression and metastases [11–14]. A recent analysis of The Cancer Genome Atlas (TCGA) showed that multiple cancers, including breast cancer, have increased MMP mRNA expression compared to control normal tissue for a given patient (Nguyen, manuscript in preparation). The Py8119 tumors used in this study represent the highly invasive triple negative breast cancer that have elevated levels of MMP-2 and MMP-9 activity. Spontaneous murine MMTV-polyomavirus middle T tumors [17, 20] and multiple cell line derived tumors, both human and murine [30, 31], can activate ACPPs, providing evidence for the broad clinical applications of this agent. While ACPPD-Gd accumulation is enzyme driven and is only limited by agent availability, receptor-targeted agents may require individualization for different tumor types, and accumulation may be limited by ligand avidity and receptor density.

This study was done to detect whether a large, molecularly targeted probe could conceivably aid in the diagnosis of smaller tumors in this animal model more than the water-soluble small molecular weight agents currently available in the clinic. Since the interpreting radiologists never saw the animals, and the tumors were tracked over time, we were able to obtain a receiver operator characteristic curve and determine detection thresholds for small tumors. This experimental paradigm enabled us to perform a preclinical study to gain insight into the potential performance of this agent at later stages in translational development, and to guide future work.

The study has several limitations. Working in an allograft model makes experimental timing more tractable and the model more consistent, but it is well recognized that allografts are not an optimal model for human tumors. Second, like all molecularly targeted probes of its size, our molecule is not entirely specific. This is not necessarily a disadvantage, and part of this study was done to determine whether these molecules might nevertheless have an advantage over the current clinically available gadolinium chelates. We were able to improve upon prior work by demonstrating utility of the probe with a clinical strength 3T magnet. This is important because gadolinium contrast agents have different physical properties at 3T than they do at 7T. Our technique provides a tractable and practical model to test and compare other contrast agents in a more clinically relevant model than simple region of interest or standardized uptake values. Future studies will examine whether such an integrated approach is more predictive of how well an agent will perform in the clinic.

In conclusion, ACPPD-Gd provided significantly greater tumor enhancement on T1w MR imaging at 3 Tesla compared to the equilibrium phase of standard Gd-chelates aiding in the detection of tumors in this mammary tumor model at clinically relevant fields strengths and scan times. Future work will expand upon the findings established in this study and will address specificity in a model that produces both benign and malignant tumors to more closely resemble the clinical setting.

## Supporting Information

S1 File MR imaging locations and transport. (DOCX)

S2 File Animal Food. (DOCX)

S3 File Structural environment for animal housing. (DOCX)

S4 File NC3Rs ARRIVE Guidelines Checklist. (PDF)
