# Supplementary material for: Tumor Detection at 3 Tesla with an Activatable Cell Penetrating Peptide Dendrimer (ACPPD-Gd), a T1 Magnetic Resonance (MR) Molecular Imaging Agent
Source: PLoS One. 2015 Sep 3;10(9):e0137104. doi: 10.1371/journal.pone.0137104 (PMC4559389; doi:10.1371/journal.pone.0137104)
Supplement: S1 File — (DOCX) [file pone.0137104.s001.docx]

**S1. MR imaging locations and transport: 3T at Bydder Lab.** Animals that are going between the Tsien lab/vivarium space and Bydder 3T MRI will be transported by lab personnel in personal vehicles per IACUC policy 33.0, as due to the heavy demand and limited availability of time on these instruments, the hours of these experiments may be outside the normal hours that ACP personnel are working. ACP transfer paperwork will be submitted online for each request, animals will only be moved with ACP approval. In brief, mice will be moved in disposable primary mouse cages provided by ACP, which will be placed in a secured secondary plastic container to prevent escape and covered by a cloth. The cage will be secured in the vehicle to prevent jarring, tipping and escape. Mice will only be moved in personal vehicles with ACP required environmental controls (ie. air conditioning, heating). Mice will not be placed in the trunk of the vehicle.
