# Supplementary material for: Tumor Detection at 3 Tesla with an Activatable Cell Penetrating Peptide Dendrimer (ACPPD-Gd), a T1 Magnetic Resonance (MR) Molecular Imaging Agent
Source: PLoS One. 2015 Sep 3;10(9):e0137104. doi: 10.1371/journal.pone.0137104 (PMC4559389; doi:10.1371/journal.pone.0137104)
Supplement: S2 File — (DOCX) [file pone.0137104.s002.docx]

**S2. Animal Food**: Immune competant animals will be fed routinely with Harlan/Teklad 2018. Cages will be labelled as such.
