# Supplementary material for: Tumor Detection at 3 Tesla with an Activatable Cell Penetrating Peptide Dendrimer (ACPPD-Gd), a T1 Magnetic Resonance (MR) Molecular Imaging Agent
Source: PLoS One. 2015 Sep 3;10(9):e0137104. doi: 10.1371/journal.pone.0137104 (PMC4559389; doi:10.1371/journal.pone.0137104)
Supplement: S3 File — (DOCX) [file pone.0137104.s003.docx]

**S3. Structural environment for animal housing**: The primary enclosure will be the standard mouse cage issued by the animals' original vivarium where they were housed until the time of the experiment. The cage (singular-as one cage will be kept in this room at a time) will be inside a Baker Steri Guard III hood with a 12 hour light cycle. A time controlled lighting system will be used to ensure the appropriate diurnal cycle. The windows of this room have been blacked out for imaging purposes. This is typically a very quiet room. No more than two people may comfortably work together in this room at a time. There is one door and zero traffic. Temperature/circulation A work order has been submitted to physical plant denoting the temperature (64-79ºF) and humidity (30-70%) restrictions for a mouse room. We have purchased a humidity/temperature pen with memory to verify that the room has remained within these restraints. With the hood, any recycled air (less than 50%) is carried through hepa filters. These filters are changed every three months. The animals will be checked every six hours as necessitated by the experimental imaging protocol. Humidity and temperature highs and lows will be noted every 24 hours. The animals will be kept no longer than 72 hours negating the need for cage changes. An extra clean cage will be kept in the room at all times, however, should a water bottle cause an unexpected flood. These observations will be noted daily on a form similar to a vivarium room status report.
